# Enhanced Efficacy of a TLR3 Agonist Delivered by Cowpea Chlorotic Mottle Virus Nanoparticles

**DOI:** 10.1002/smsc.202300314

**Published:** 2024-04-25

**Authors:** Eunkyeong Jung, Anahid Foroughishafiei, Young Hun Chung, Nicole F. Steinmetz

**Affiliations:** ^1^ Department of NanoEngineering University of California San Diego 9500 Gilman Dr. La Jolla CA 92093 USA; ^2^ Department of Bioengineering University of California San Diego 9500 Gilman Dr. La Jolla CA 92093 USA; ^3^ Department of Radiology University of California San Diego 9500 Gilman Dr. La Jolla CA 92093 USA; ^4^ Center for Nano‐ImmunoEngineering University of California San Diego 9500 Gilman Dr. La Jolla CA 92093 USA; ^5^ Shu and K.C. Chien and Peter Farrell Collaboratory University of California San Diego 9500 Gilman Dr. La Jolla CA 92093 USA; ^6^ Center for Engineering in Cancer Institute of Engineering in Medicine University of California San Diego 9500 Gilman Dr. La Jolla CA 92093 USA; ^7^ Moores Cancer Center University of California University of California San Diego 9500 Gilman Dr. La Jolla CA 92093 USA; ^8^ Institute for Materials Discovery and Design University of California San Diego 9500 Gilman Dr. La Jolla CA 92093 USA

**Keywords:** cancer immunotherapy, cowpea chlorotic mottle virus, drug delivery, intratumoral immunotherapy, TLR3 agonist, virus like particles

## Abstract

Intratumoral immunotherapies are those that are administered directly into a tumor to remodel the local tumor microenvironment and stimulate systemic anti‐tumor immunity. Small molecule toll‐like receptor (TLR) agonists are undergoing development as intratumoral immunotherapies, and here the TLR3 agonist poly(I:C) is considered. Because small molecule therapeutics often suffer rapid washout effects and ineffective immune cell uptake, poly(I:C) is encapsulated into nanoparticles derived from cowpea chlorotic mottle virus (CCMV). The particles (but not the separate components) stimulate the activity of macrophages in vitro and are able to reduce tumor growth and prolong survival in mouse models of colon cancer and melanoma. CCMV‐poly(I:C) is also combined with oxaliplatin and found the combination therapy to be even more potent, strongly inhibiting tumor growth and increasing survival. The analysis of immune markers reveals that CCMV‐poly(I:C) VLPs with oxaliplatin promoted the infiltration and activation of CD4^+^ and CD8^+^ cells and the production of IL‐4 and IFN‐γ, indicating a synergistic immunogenic effect. The combined treatment also enhances the rate of apoptosis and immunogenic cell death (ICD). The data support the development of combination therapies involving immunomodulatory plant virus nanoparticles and antineoplastic drugs to attack tumors directly and via the activation of innate and adaptive immune responses.

## Graphical abstract

This study investigates intratumoral immunotherapy using a TLR3 agonist, poly(I:C), encapsulated in cowpea chlorotic mottle virus (CCMV) nanoparticles. These nanoparticles effectively stimulate immune responses, reducing tumor growth and enhancing survival in colon cancer and melanoma mouse models. Combining CCMV‐poly(I:C) with oxaliplatin further improves efficacy, inducing immune cell infiltration, and cytokine production, suggesting promising combination therapies for cancer treatment.

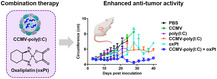

## 1 Introduction

Cancer immunotherapy is a promising collection of treatments that harness the ability of the immune system to fight cancer. In contrast to surgery, radiotherapy and chemotherapy, each of which requires a radical external intervention, immunotherapy is based on enhancing natural anti‐cancer mechanisms, particularly those that are suppressed by aggressive tumors.^[^ ^1^ ^]^ Cancer cells produce tumor‐associated and neoantigens that are recognized by the immune system, allowing the tumors to be targeted and eradicated by tumor‐infiltrating lymphocytes (TILs). However, many cancer cells also possess the ability to create an immunosuppressive tumor microenvironment (TME) by blocking the infiltration, activation and effector functions of immune cells, evading immunosurveillance, and stimulating pathways that recruit immunosuppressive regulatory T (T_reg_) cells and tumor‐associated macrophages.^[^ ^2^ ^]^ The immunosuppressive TME results in so‐called “cold” tumors that tend to respond poorly to immunotherapy. The administration of systemic immunomodulators such as cytokines and checkpoint inhibitors can reverse this immunosuppressive state, but they cause severe side effects.^[^ ^3^ ^]^

One approach to overcome these challenges is intratumoral therapy, in which a therapeutic is introduced directly into the tumor to induce a local, antigen‐specific anti‐tumor immune response.^[^ ^4^ ^]^ The therapeutic agent is generally an immunostimulatory adjuvant, which converts the immunosuppressive TME to an immunostimulated phenotype, thus restoring the normal cancer immunity cycle and allowing the natural immune response to deal with “hot” tumors.^[^ ^5^, ^6^ ^]^ Immunologically cold tumors can be made hot by targeting endosomal Toll‐like receptors (TLRs), specifically TLR3, TLR7, TLR8, and TLR9, within antigen‐presenting cells (APCs).^[^ ^7^ ^]^ In the clinic, the dermal application of imiquimod (TLR7 agonist) and resiquimod (TLR7/8 agonist) has confirmed the ability of such drugs to induce a local immune response against cutaneous tumors while limiting systemic exposure and side effects,^[^ ^8^ ^]^ and the approval of tamilogene laherparepvec (T‐VEC), an oncolytic herpesvirus that expresses granulocyte‐macrophage colony‐stimulating factor (GM‐CSF), has confirmed that intratumoral immunotherapy is suitable for the treatment of melanoma.^[^ ^9^ ^]^

Here we focused polyinosinic:polycytidylic acid (poly(I:C)), which resembles the structure of dsRNA and agonizes TLR3. TLR3 recognizes double stranded RNA to activate innate immune cells (macrophages, dendritic cells) to become APCs leading to CD4^+^ T cell responses to switch from Th2 to Th1 while boosting the CD8^+^ T cell response and inhibiting T_reg_ cells.^[^ ^10^ ^]^ The intratumoral application of free poly(I:C) is hampered by rapid washout effects and limited cell uptake—to overcome these shortcomings and control tissue diffusivity and enhance cell uptake, we opted to encapsulate poly(I:C) into nanoparticles derived from cowpea chlorotic mottle virus (CCMV).

Several plant virus nanoparticles are undergoing development for cancer immunotherapy, this includes papaya mosaic virus (PapMV),^[^ ^11^ ^]^ cowpea mosaic virus (CPMV),^[^ ^12^ ^]^ and potato virus X (PVX).^[^ ^13^ ^]^ In the latter case, we showed that the PVX nanoparticles have immunomodulatory effects after intratumoral administration and combination of PVX and chemotherapy doxorubicin resulted in potent anti‐tumor immunity. This supports earlier studies showing that immunotherapy and chemotherapy can achieve synergistic effects that benefit patient outcomes. The potent effect of PapMV was attributed to its encapsulated RNA, which was shown to activate signaling through TLR7.^[^ ^14^ ^]^ Further, we found that CPMV is a potent anti‐tumor agent and its potency is attributed to its signaling through multiple TLRs; the proteinaceous capsid is recognized by TLR2 and TLR4 while the RNA is recognized by TLR7.^[^ ^15^, ^16^ ^]^ In contrast, CCMV alone did not exhibit anti‐tumor efficacy; however, when loaded with CpG oligodeoxynucleotides it was shown to induce the activity of tumor‐associated macrophages resulting in potent anti‐tumor immune responses in tumor mouse models.^[^ ^17^ ^]^ Given that CCMV has proven useful for the delivery of TLR agonists but has not yet been tested in the context of combination therapy, here we developed CCMV particles loaded with the TLR3 agonist poly(I:C) and combined it with oxaliplatin, an antineoplastic drug.^[^ ^18^ ^]^ The underlying hypothesis is that the chemotherapy induces cancer cell death to release tumor associated and neoantigens to be processed by innate immune cells recruited and activated by the TLR3 agonist, therefore resulting in improved therapy success. We tested this hypothesis in a mouse model of colon cancer and investigated the underlying immunological mechanism through a combination of chemo/cytokine analysis, immunological cell profiling, and tumor histology imaging.

## 2 Results and Discussion

### 2.1 Preparation and Characterization of CCMV‐poly(I:C) Virus Like Particles (VLPs)

**Figure 1:**
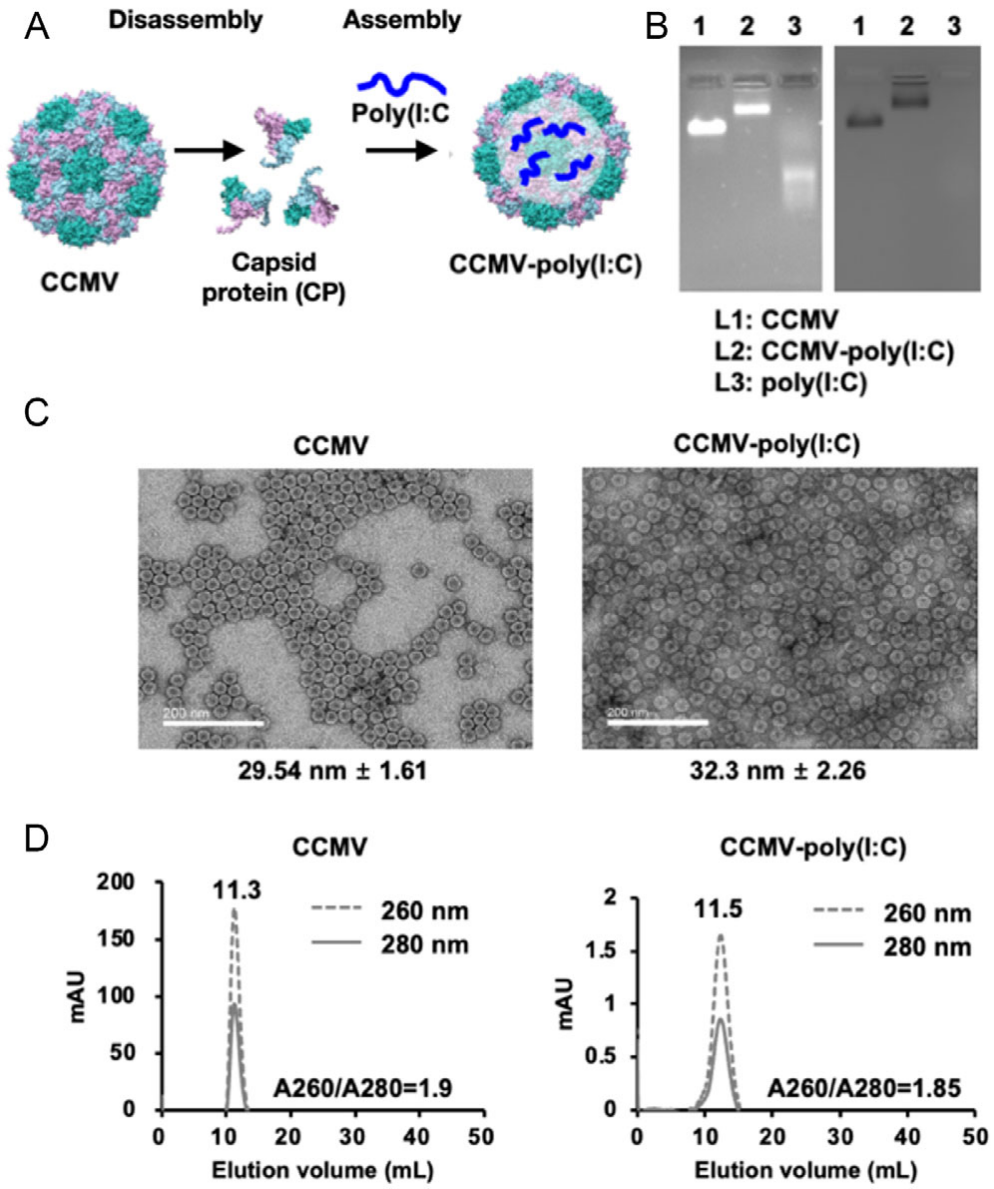
Characterization of CCMV‐poly(I:C) VLPs. A) Schematic illustration showing the preparation of CCMV‐poly(I:C). CCMV was disassembled into capsid proteins (CPs) at pH 7.5 in the presence of Ca^2+^ and Mg^2+^ ions, before reassembly with poly(I:C) at pH 4.2. B) Agarose gel electrophoresis. Left panel shows RNA stained with 2% GelRed under UV light. Right panel shows protein stained with Coomassie Brilliant Blue under white light. C) TEM images of CCMV and CCMV‐poly(I:C) VLPs (Scale bar = 200 nm). Average size was determined using ImageJ. Data are means ± standard deviations (*n* = 5). D) SEC profiles of CCMV and CCMV‐Poly(I:C) VLPs.

CCMV‐poly(I:C) virus‐like particles (VLPs) were prepared by the disassembly of native CCMV into capsid proteins and viral RNA, followed by the precipitation of the RNA.^[^ ^19^ ^]^ The capsid proteins and poly(I:C) were then mixed at a mass ratio of 6:1 and assembled in an acidic buffer (**Figure** **1**A).^[^ ^20^ ^]^ The number of poly(I:C) molecules encapsidated in CCMV particles was calculated as previously described,^[^ ^17^, ^21^ ^]^ indicating that each particle contained 45 poly(I:C) molecules. The loading efficiency was 32%, which is calculated using the following equation: Amount of drug loaded in CCMV/Amount of drug added × 100%. Native agarose gel electrophoresis indicated CCMV‐poly(I:C) particles were intact with electrophoretic mobility comparable to that of native CCMV; RNA (GelRed) and protein bands (Coomassie) co‐localized and there was no evidence of free poly(I:C) (Figure 1B). The size and morphology of the particles were determined by TEM. The diameter of the CCMV‐poly(I:C) particles was 32.3 ± 2.26 nm, slightly larger than native CCMV at 29.54 ± 1.61 nm (Figure 1C). The purity and integrity of CCMV‐poly(I:C) were also confirmed by the unique SEC peak at an elution volume of ∼11 mL for both particles (Figure 1D). The A260/280 ratios of the CCMV‐poly(I:C) and CCMV peaks were ∼1.85 and ∼1.9, which is consistent with intact CCMV.

### 2.2 In Vitro Immunomodulatory Properties of CCMV‐poly(I:C)

**Figure 2:**
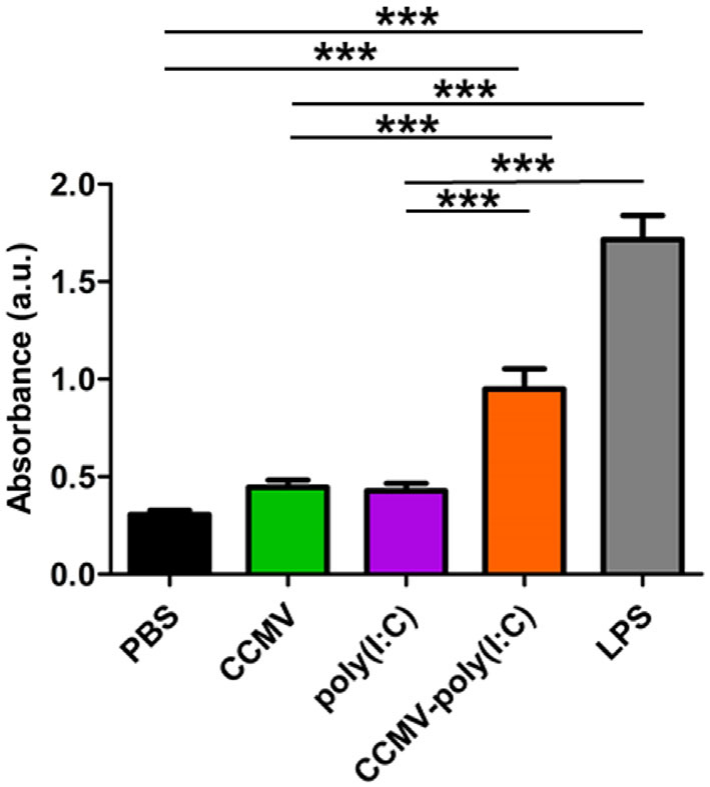
Immunogenicity of CCMV‐poly(I:C) VLPs in vitro. Activation of RAW‐Blue following treatment with CCMV, poly(I:C) or CCMV‐poly(I:C), compared to LPS as a positive control and PBS as a negative control. Values are means ± standard deviations (*n* = 7). Statistical significance was determined by one‐way ANOVA with a post hoc Tukey's HSD test (***p < 0.001).

The immunomodulatory properties of CCMV‐poly(I:C) VLPs were initially tested in vitro using RAW‐Blue cells and a Quanti‐Blue assay. SEAP levels in cells treated with CCMV and free poly(I:C) did not differ significantly from the PBS‐treated control, indicating no immunomodulatory effects of CCMV alone, which is consistent with our previous observations.^[^ ^22^ ^]^ The lack of immune stimulation of the RAW‐Blue cells by free poly(I:C) may be explained by inefficient cell uptake and trafficking. In stark contrast, CCMV‐poly(I:C) VLPs increased the SEAP levels by 3.1‐fold compared to PBS, 2.3‐fold compared to CCMV, and 2.2‐fold compared to free poly(I:C) (**Figure** **2**). This result confirmed the in vitro immunomodulatory properties of CCMV‐poly(I:C). Similarly, in our previous work CCMV‐ODN1826 particles were shown to activate RAW‐Blue cells although in this case the pathway involved was TLR9.^[^ ^17^ ^]^ We note however that additional assays could be performed to delineate whether the immunomodulatory properties are solely conferred by poly(I:C) engaging and agonizing TLR3 or whether other receptors are engaged as well.

### 2.3 Enhanced Therapeutic Efficacy of CCMV‐poly(I:C) in Combination with Oxaliplatin

**Figure 3:**
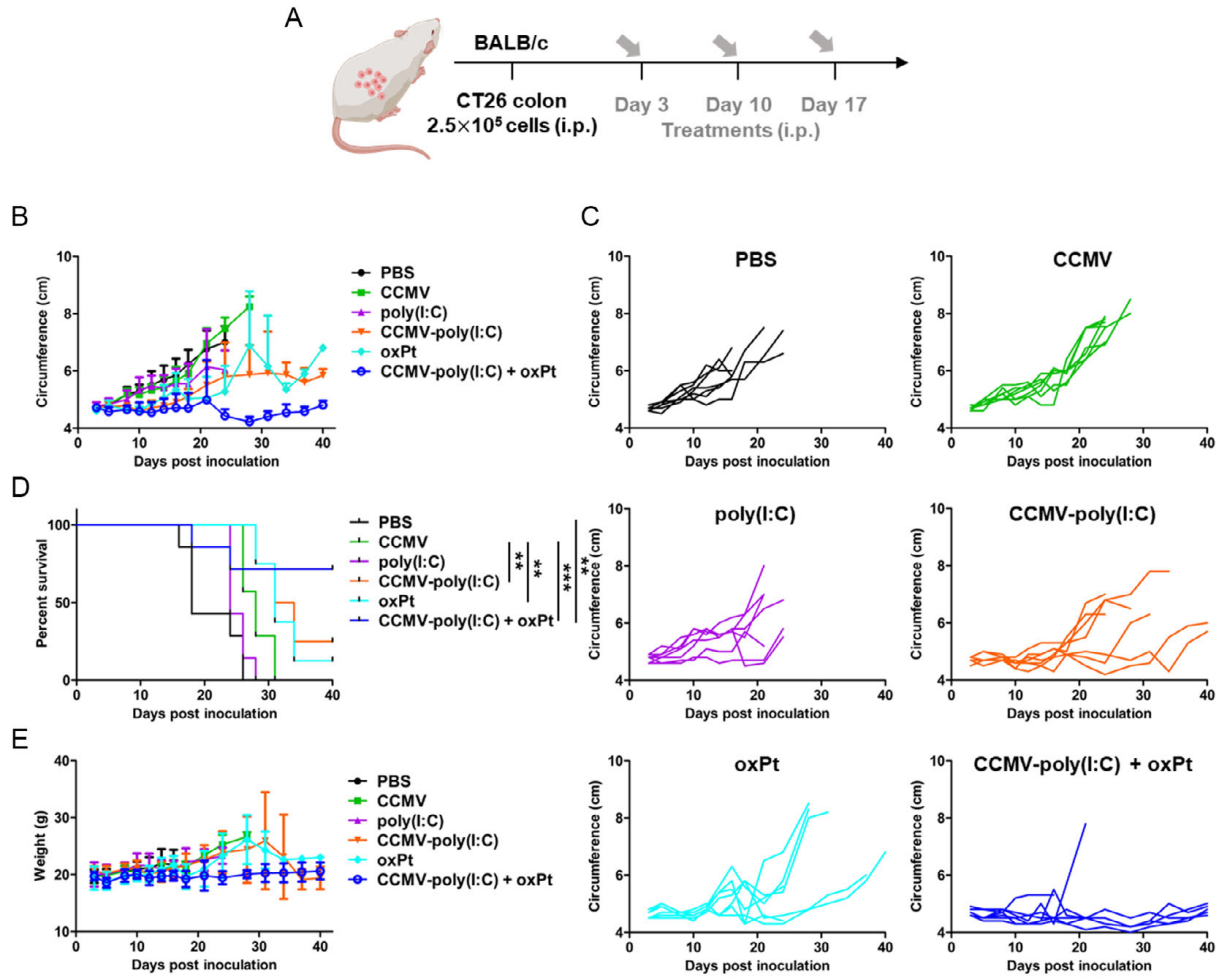
Enhanced therapeutic efficacy of CCMV‐poly(I:C) VLPs plus oxaliplatin in mice with CT26 tumors induced by i.p. inoculation. A) Schematic illustration of the experimental timeline. CT26 cells were inoculated i.p. into female BALB/c mice (*n* = 7 per group). The tumor‐bearing mice were then treated i.p. with CCMV‐poly(I:C) and/or oxPt as well as the separate CCMV and poly(I:C) components on days 3, 10 and 17. Gray arrows indicate the treatment days. B) Tumor growth curves. Data are means ± standard deviations (*n* = 7). Statistical significance was determined by one‐way ANOVA with a post hoc Tukey's HSD test (***p < 0.001, **p < 0.01). C) Tumor growth kinetics of individual mice. D) Survival rates of mice. The results were compared using the log‐rank (Mantel–Cox) test (***p < 0.001, **p < 0.01). E) Body weights of mice. Mouse image from BioRender.com.

To investigate the therapeutic efficacy of CCMV‐poly(I:C) alone and combined with oxaliplatin, we assessed the effect of each treatment in vivo using the CT26 model of colon cancer. Mice with tumors induced by i.p. inoculation with CT26 cells were assigned to six treatment groups as follows: PBS (negative control), CCMV, poly(I:C), CCMV‐poly(I:C), oxaliplatin (oxPt), or CCMV‐poly(I:C) + oxPt. CCMV alone did not inhibit tumor growth compared to the PBS control, whereas free poly(I:C) had a slight inhibitory effect, albeit not statistically significant (**Figure** **3**A). In contrast, CCMV‐poly(I:C) VLPs significantly inhibited tumor growth and also slightly increased the survival rate compared to mice treated with CCMV or poly(I:C) alone, as did the free oxPt treatment. Similarly, separate treatments with CCMV and ODN1826 had a minimal impact on CT26 tumors but the CCMV‐ODN1826 encapsulated VLPs had a significant effect.^[^ ^17^ ^]^ However, the most potent outcome was achieved by the combination of CCMV‐poly(I:C) and oxPt, which not only significantly inhibited tumor growth but also had a remarkable effect on survival, with 75% of the mice in this group surviving after 40 days (Figure 3B–D). The body weight of CT26‐bearing mice treated with PBS, CCMV or poly(I:C) increased due to the growth of the tumor, whereas there was no significant gain or loss of body weight in the CCMV‐poly(I:C) + oxPt group (Figure 3E). This is reminiscent of the effect achieved by combining PVX nanoparticles with doxorubicin, which enhanced the efficacy compared to the individual treatments in a B16F10 melanoma model but only when the VLPs and drug were mixed, not when they were physically joined by conjugation, indicating the two components act synergistically against different targets.^[^ ^13^ ^]^

**Figure 4:**
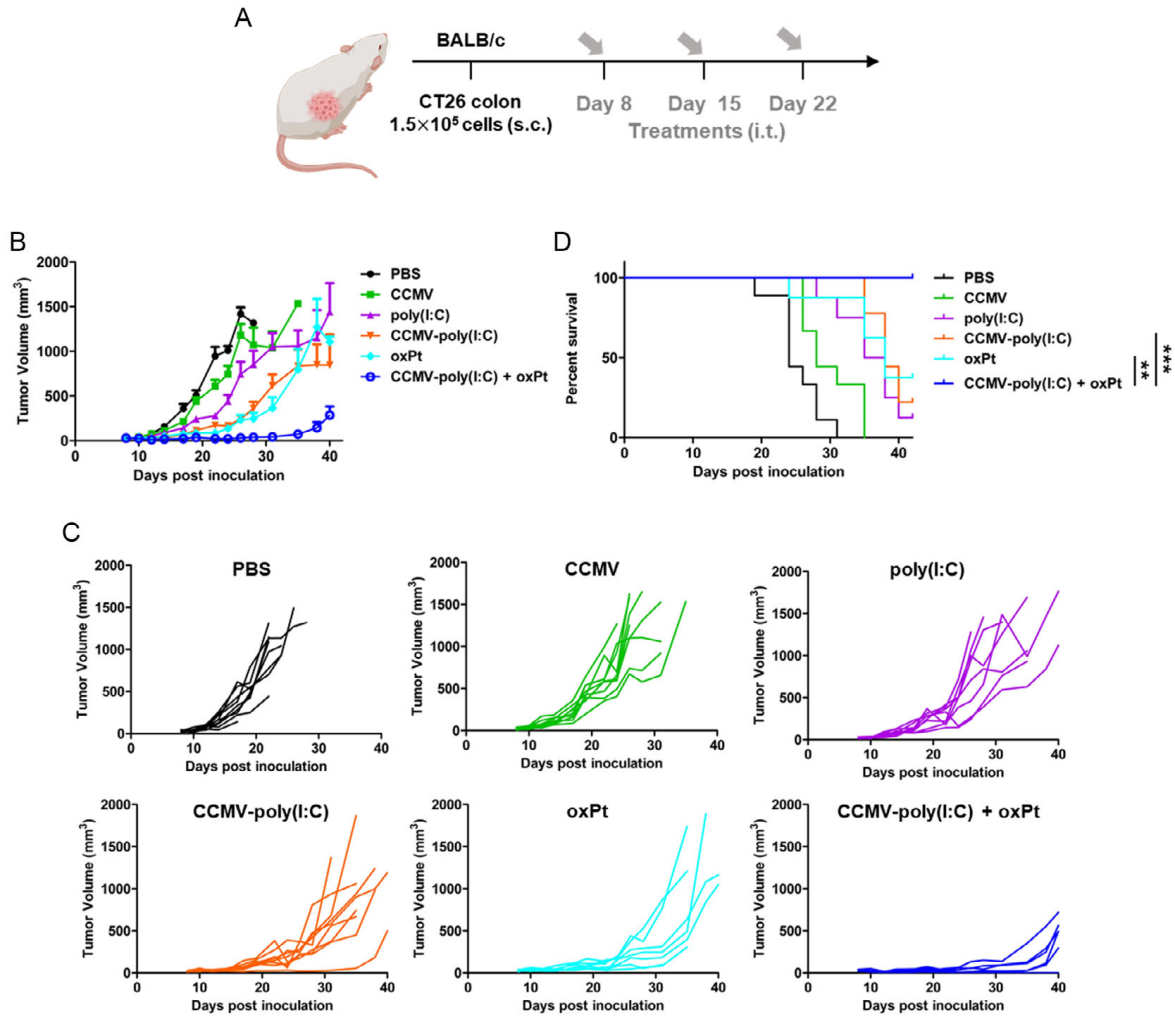
Enhanced therapeutic efficacy of CCMV‐poly(I:C) VLPs with oxaliplatin in mice bearing s.c. CT26 tumors. A) Schematic illustration of experimental timeline. CT26 cells were inoculated s.c. into the left flank of BALB/c mice (*n* = 8 per group). The tumor‐bearing mice were treated i.t. with CCMV‐poly(I:C) and oxPt once the tumor volume reached ~30 mm^3^. B) Tumor growth curves. Data are means ± standard deviations (*n* = 8). The results were compared by one‐way ANOVA with a post hoc Tukey's HSD test (***p < 0.001, **p < 0.01). C) Tumor growth kinetics of individual mice. D) Survival curves. The results were compared using the log‐rank (Mantel–Cox) test (***p < 0.001). Mouse image from BioRender.com.

The results observed in the CT26 i.p. model were replicated in the CT26 s.c. model (**Figure** **4**A). In the PBS and CCMV groups, the tumors grew 47.5‐fold and 52.4‐fold, respectively, by day 26. In the poly(I:C) group, tumor growth was suppressed but not to a statistically significantly extent (30‐fold growth by day 26). In the CCMV‐poly(I:C) and oxPt groups, the tumors grew to similar volumes of ∼242 and ∼233 mm^3^, respectively. However, in the CCMV‐poly(I:C) + oxPt group the volume of the tumors on day 26 was just 30 mm^3^ (Figure 4B,C). The CCMV‐poly(I:C) + oxPt treatment also had the most significant effect on survival, with 100% of the mice in this group remaining alive after 40 days (Figure 4D).

### 2.4 Enhanced Cytokine Stimulation and T Cell Infiltration as a Result of CCMV‐poly(I:C) in Combination with Oxaliplatin Therapy

**Figure 5:**
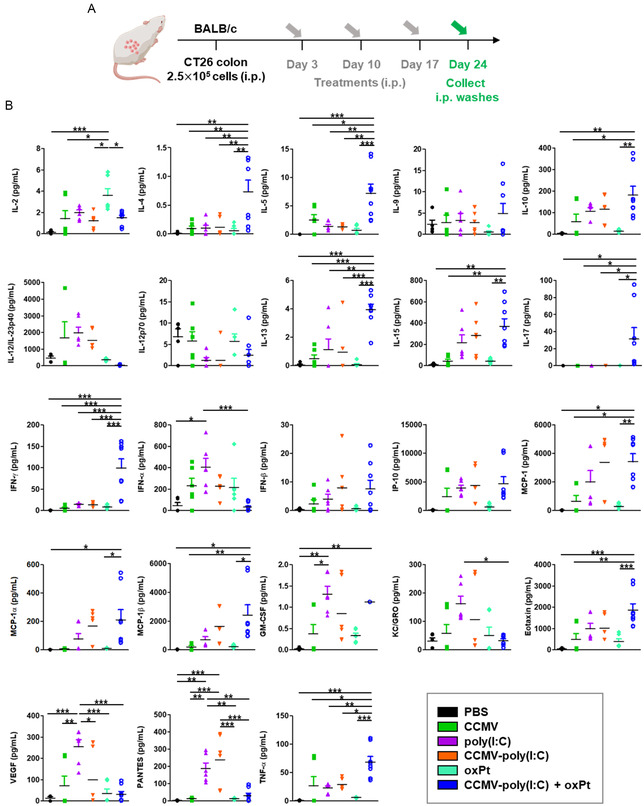
Stimulation of cytokine production by CCMV‐poly(I:C) VLPs with oxaliplatin in mice bearing CT26 tumors induced by i.p. inoculation. A) Experimental timeline for treatment and the collection of i.p. washes. B) The level of pro‐inflammatory cytokines and chemokines measured using a mesoscale discovery (MSD) assay. Data are means ± standard deviations (*n* = 6). Statistical significance was determined by one‐way ANOVA with a post hoc Tukey's HSD test (***p < 0.001, **p < 0.01, *p < 0.05). Mouse image from BioRender.com.

The underlying mechanisms of the observed in vivo responses were determined by the analysis of i.p. washes and splenocytes. We used an MSD assay to measure the levels of cytokines and chemokines in i.p. washes collected from immunized mice (**Figure** **5**). As shown in Figure 5B, PBS or CCMV‐treated tumors did not show any difference in the production of cytokines or chemokines in i.p. washes. However, CCMV‐poly(I:C) or CCMV‐poly(I:C) + oxPt showed considerable difference in the level of cytokines or chemokines compared to CCMV and poly(I:C) alone. Interestingly, CCMV‐poly(I:C) + oxPt notably increased some cytokines and chemokines, including IL‐4, IL‐5, IL‐13, IL‐15, IFN‐γ, IP‐10, MCP‐1, MCP‐1α, MCP‐1β, Eotoxin, and TNF‐α.

**Figure 6:**
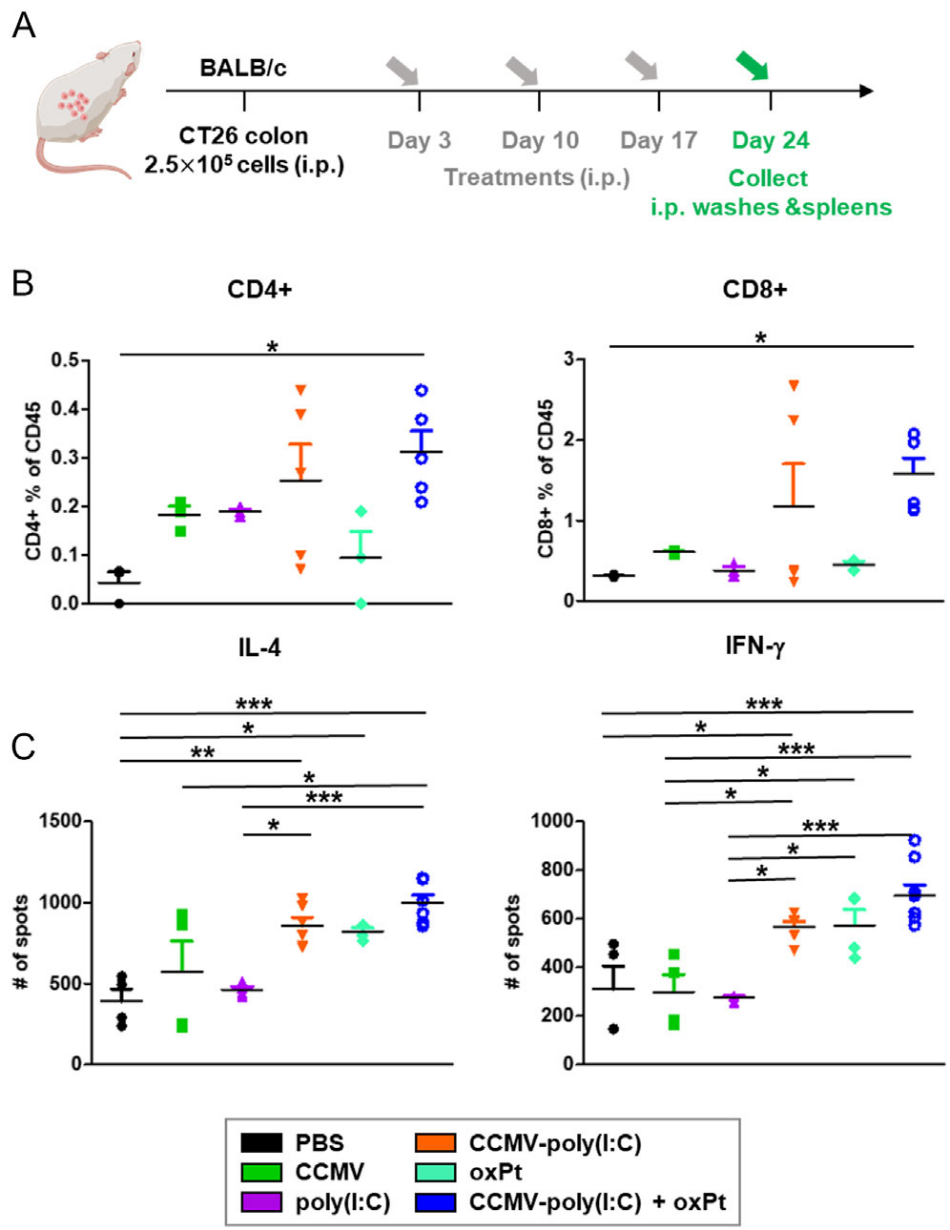
T cell analysis in response to CCMV‐poly(I:C) VLPs with oxaliplatin treatment in mice bearing i.p. CT26 tumors. A) Experimental timeline for treatment and the collection of i.p. washes and spleens on day 24. B) The population of CD4^+^ and CD8^+^ cells in the i.p. washes. C) The level of IL‐4 and IFN‐γ produced by splenocytes from immunized mice. Data are means ± standard deviations (*n* = 6). Statistical significance was determined by one‐way ANOVA with a post hoc Tukey's HSD test (***p < 0.001, **p < 0.01, *p < 0.05). Mouse image from BioRender.com.

The same washes were analyzed by flow cytometry to detect activated CD4^+^ and CD8^+^ cells (**Figure** **6**). Cells were pre‐gated on live, single lymphocytes and defined by CD45^+^ and CD3^+^ expression before further gating to identify CD4^+^ and CD8^+^ T cells. Although CCMV‐poly(I:C) increased the infiltration of T cells compared to CCMV and poly(I:C) alone, CCMV‐poly(I:C) + oxPt induced the most prolific infiltration of CD4^+^ and CD8^+^ cells (Figure 6B). We also used an ELISPOT assay to evaluate the production of IL‐4 and IFN‐γ from splenocytes, which play major roles in regulating immune responses (Figure 6C). In agreement with the MSD and flow cytometry data, CCMV‐poly(I:C) + oxPt enhanced the levels of IL‐4 and IFN‐γ more than any other treatment, confirming the synergistic immunogenic effect of the immunomodulatory VLPs and oxPt. It should be noted that T cell depletion studies and analysis of T cell specificity are required to further delineate the role of T cell responses and whether the treatment is T cell dependent.

Here we focused on assaying changes of immune cells and cyto/chemokines within the TME (i.p. space) and spleen—future work also should assess systemic cyto/chemokines profiling as well as distribution of poly(I:C) delivered free as soluble injection of packaged into CCMV. The underlying hypothesis is that the nanoparticle‐formulation, CCMV‐poly(I:C), leads to enhanced tumor retention thus reducing systemic exposure and associated inflammatory toxicity. In other works, we have demonstrated tumor retention of plant virus nanoparticle formulations with minimal leaching^[^ ^23^ ^]^—these assays need to be applied toward the pharmacological and toxicology testing of the proposed CCMV‐poly(I:C) immunotherapy candidate.

### 2.5 Enhanced Immunogenic Cell Death Effect of CCMV‐poly(I:C) in Combination with Oxaliplatin

**Figure 7:**
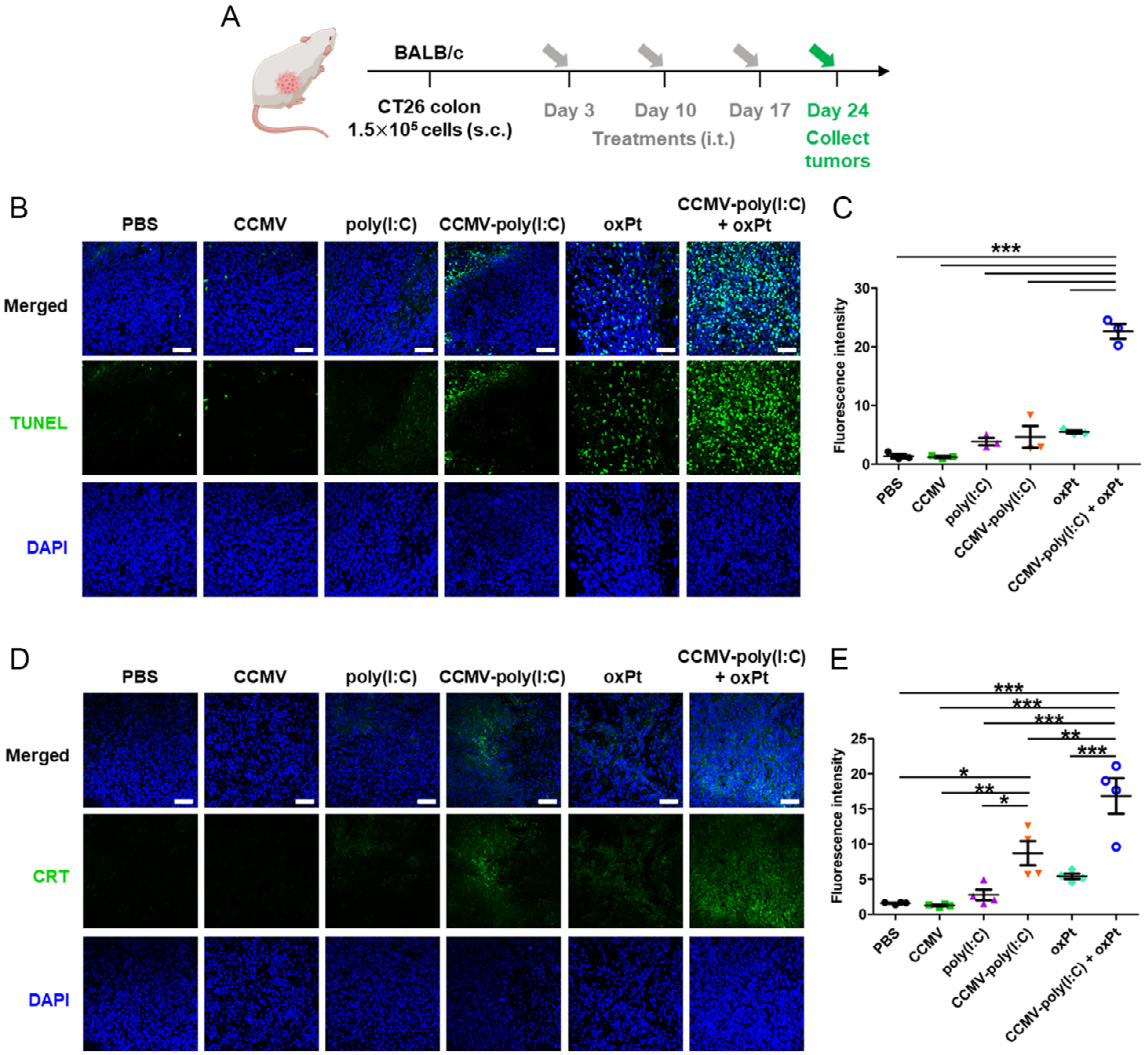
Histological evaluation of colon tumor tissues treated with CCMV‐poly(I:C) VLPs and oxPt. A) Experimental timeline. Gray arrows indicate treatment with CCMV‐poly(I:C) VLPs and oxPt. CT26 s.c. tumors were harvested on day 24. B) TUNEL staining of tumor tissues (scale bar = 20 μm). C) Quantitative analysis of fluorescence from TUNEL‐positive cells using ImageJ software. D) Calreticulin (CRT) exposure staining and E) quantification of fluorescence intensity. Data are means ± standard deviations (*n* = 4). Statistical significance was determined by one‐way ANOVA with a post hoc Tukey's HSD test (***p < 0.001, **p < 0.01, *p < 0.05). Mouse image from BioRender.com.

We verified the anti‐tumor mechanism of CCMV‐poly(I:C) + oxPt histologically using mice bearing CT26 tumors induced by s.c. inoculation. Mice were treated i.t. three times with CCMV, poly(I:C), CCMV‐poly(I:C) or oxPt. On day 24, tumor‐bearing mice were euthanized, and tumors were collected and stained using the TUNEL assay to detect apoptotic cells or an anti‐CRT antibody to detect ICD events (**Figure** **7**A). TUNEL fluorescence was not detected in tumor tissues treated with PBS or CCMV, indicating no anti‐tumor activity, whereas the CCMV‐poly(I:C) treatment enhanced apoptosis in the tumors compared to free poly(I:C) at the same dose (Figure 7B,C). Apoptosis was enhanced further in the CCMV‐poly(I:C) + oxPt combined treatment compared to the CCMV‐poly(I:C) or oxPt treatments alone, with CCMV‐poly(I:C) + oxPt showing 5‐fold and 5.2‐fold increase in apoptosis vs. CCMV‐poly(I:C) or oxPt, respectively. The histology analysis thus confirms the synergistic anti‐tumor effect.

oxPt causes immunogenic cell death (ICD),^[^ ^24^ ^]^ a form of cell death that stimulates the immune system through release and presentation of danger signals or damage‐associated molecular patterns (DAMPs), such as ATP, CRT, HMGB1, and heat‐shock proteins.^[^ ^25^ ^]^ To probe whether DAMPs are involved, we imaged CRT. While CCMV‐poly(I:C) slightly increased the CRT exposure compared to CCMV and poly(I:C), the combination of CCMV‐poly(I:C) + oxPt significantly increased the level of CRT exposure, which is 2.7‐fold and 2.5‐fold increase compared to CCMV‐poly(I:C) or oxPt, respectively. (Figure 7D,E).

## 3 Conclusion

We have demonstrated that the TLR3 agonist poly(I:C) can be encapsulated within CCMV particles (CCMV‐poly(I:C) VLPs) and that the resulting formulation is stable and intact in vitro and in vivo. The CCMV‐poly(I:C) VLPs were immunomodulatory in vitro, as determined by their ability to stimulate the production of SEAP by RAW‐Blue macrophages, whereas the components supplied separately had no significant effect. The therapeutic impact of CCMV‐poly(I:C) alone and combined with oxaliplatin was assessed in vivo using two CT26 colon carcinoma models. CCMV alone had no effect whereas poly(I:C) and oxaliplatin were able to induce weak to moderate immune responses that slightly inhibited tumor growth and/or improved survival. The CCMV‐poly(I:C) VLPs were more efficacious than either of their components, but the most potent effects in terms of both tumor inhibition and survival in all three models were achieved by the combination of CCMV‐poly(I:C) VLPs and oxaliplatin. The underlying mechanism involves the infiltration and activation of CD4^+^ and CD8^+^ cells and the production of IL‐4 and IFN‐γ in the TME, indicating a synergistic immunogenic effect. The combined treatment also enhanced the rates of apoptosis and ICD, confirming the more potent anti‐tumor response. Additional research is required to understand the synergistic mechanisms in more detail, but our results support earlier studies based on CPMV, PapMV and PVX indicating that the intratumoral injection of plant VLPs induces a localized immune response that involves the attraction of tumor infiltrating lymphocytes and the secretion of cytokines and chemokines that convert the immunosuppressive TME into an immunostimulated phenotype. The interplay between innate and adaptive immunity results in a systemic response and the establishment of immune memory to ensure that residual cancer cells are eliminated. While the VLPs act upon the immune system, the co‐administration of a separate antineoplastic drug attacks the cancer cells directly, weakening them and making them less likely to evade the immune response. The two components therefore act synergistically by acting against different targets, increasing the overall potency of the anti‐tumor effect.

## 4 Experimental Section

4.1

4.1.1

##### Preparation of CCMV‐poly(I:C) VLPs

CCMV was produced as previously described.^[^ ^21^ ^]^ Briefly, black‐eyed pea No. 5 plants were mechanically infected with CCMV and leaves were harvested 14 days post‐inoculation. The leaves were homogenized, extracted with chloroform, and CCMV was purified by ultracentrifugation on a sucrose cushion. CCMV capsid proteins were acquired by overnight dialysis in disassembly buffer (0.05 m Tris‐HCl, 0.5 m CaCl_2_, 0.001 m EDTA, 0.001 m DTT, 0.0005 m PMSF, pH 7.5) and the precipitated RNA was removed by centrifugation (15 000 g, 2 h, 4 °C). The purity of the capsid proteins was determined by measuring the UV/vis absorbance ratio (A280/260 ∼1.5) before resuspending in protein buffer (0.02 m Tris‐HCl, 1 m NaCl, 0.001 m EDTA, 0.001 m DTT, 0.001 m PMSF, pH 7.2). To prepare the CCMV‐poly(I:C) VLPs, the capsid proteins were reassembled with 0.2–1 kb poly(I:C) (InvivoGen) at a 6:1 mass ratio in reassembly buffer (0.5 m Tris‐HCl, 0.5 m NaCl, 0.1 m KCl, 0.05 m MgCl_2_, 0.01 m DTT, pH 7.2). The reconstituted CCMV‐poly(I:C) particles were then stored in virus suspension buffer (0.5 m sodium acetate, 0.08 m magnesium acetate, pH 4.5) at 4 °C. The particle concentration was determined using a Pierce BCA protein kit (Thermo Fisher Scientific). The encapsulation of poly(I:C) was confirmed using a Quant‐it RiboGreen RNA assay kit (Thermo Fisher Scientific).

##### Analysis of CCMV‐poly(I:C) VLPs

The UV/vis absorbance profiles of CCMV and CCMV‐poly(I:C) were acquired using a NanoDrop spectrophotometer (Thermo Fisher Scientific). The concentration of CCMV was calculated according to Beer's Law (*A* = εbc), where A is the absorbance, ε is the molar absorptivity coefficient of 5.85 mg^−1^ cm^−1^, b is the length of the light path, and c is the concentration. The CCMV and CCMV‐poly(I:C) particles were loaded onto 1.2% w/v agarose native gels (10 μg per lane) stained with GelRed for nucleic acids and separated by electrophoresis in virus separation buffer (0.1 m sodium acetate, 1 mM EDTA, pH 5.5) for 1 h at 60 V and 4 °C.^[^ ^21^ ^]^ The gels were imaged using an AlphaImager System under UV light to detect RNA before staining with Coomassie Brilliant Blue to detect protein under white light. For transmission electron microscopy (TEM), CCMV and CCMV‐poly(I:C) were dispersed in deionized water at 100 μg mL^−1^ and dropped onto Formvar carbon film‐coated copper grids (Ted Pella) and incubated for 2 min, followed by washing twice with deionized water for 1 min. The grids were coated with 2% w/v uranyl acetate for 2 min and images were acquired using a JEOL JEM‐1400Plus microscope. The particle size was measured using ImageJ. CCMV and CCMV‐poly(I:C) size and integrity was further corroborated using fast protein liquid chromatography (FPLC) on the ÄKTA pure chromatography system (Cytiva). Particles were analyzed using a Superose 6 Increase column at a flow rate of 0.5 mL min^−1^; RNA was detected at 260 nm and protein at 280 nm.

##### Cell Cultures

RAW‐Blue cells (InvivoGen) were grown in Dulbecco's Modified Eagle's Medium (DMEM) supplemented with 10% v/v heat‐sterilized fetal bovine serum (FBS; Atlanta Biologicals) and 1% v/v penicillin/streptomycin (p/s; Thermo Fisher Scientific). Normocin (50 mg mL^−1^) and zeocin (100 mg mL^−1^) were also added according to the supplier's instructions (InvivoGen). CT26 and 4T1 cells obtained from the ATCC were grown in Roswell Park Memorial Institute (RPMI) 1640 medium and DMEM, respectively, each supplemented with 10% v/v FBS and 1% v/v p/s.

##### In Vitro Immunogenicity

RAW‐Blue cells (1 × 10^5^ cells/well) were seeded in 96‐well plates and incubated with CCMV (1 μg), poly(I:C) (0.3 μg) or CCMV‐poly(I:C) (1 μg) for 24 h. Lipopolysaccharide (LPS; InvivoGen) was used as a positive control. After the incubation, 20 μL of the supernatant was mixed with 180 μL of Quanti‐Blue solution (InvivoGen) and incubated at room temperature for 6 h. The absorbance was then measured at 630 nm using a microplate reader (Tecan).

##### In Vivo Anti‐Tumor Efficacy

Female BALB/c and C57BL/6 mice (6–7 weeks old) were purchased from the Jackson Laboratory. All animal studies were approved by and conducted according to the regulation of the Institutional Animal Care and Use Committee of the University of California, San Diego. CT26 cells were administered via the intraperitoneal (i.p.) and subcutaneous (s.c.) routes. To establish the CT26 colon cancer i.p. model, BALB/c mice were injected i.p. with 2.5 × 10^5^ CT26 cells/200 μL PBS followed by i.p. treatment with CCMV (200 μg in 200 μL PBS), poly(I:C) (∼60 μg in 200 μL PBS), CCMV‐poly(I:C) (200 μg in 200 μL PBS), oxPt (5 mg kg^−1^) or CCMV‐poly(I:C) + oxPt (doses as above) 3 days after cell inoculation. Treatments were administered three times at weekly intervals. The body circumference and weight were recorded for 40 days at 2‐day intervals and mice were euthanized when the circumference reached ∼8 cm. To establish the CT26 colon cancer s.c. model, CT26 cells (1.5 × 10^5^ cells in 15 μL PBS) were mixed with 15 μL Matrigel (Corning) and injected s.c. into the left flank of BALB/c mice. When tumors grew to ∼30 mm^3^, mice were treated intratumorally (i.t.) with CCMV (200 μg in 30 μL). The tumor volumes, calculated using the equation tumor width^2^ × tumor length)/2, and body weight were recorded for 40 days at 2‐day intervals and mice were euthanized when the tumor volume reached ∼1500 mm^3^.

##### Mesoscale Discovery (MSD) Assay

BALB/c mice were challenged i.p. with 1.5 × 10^5^ CT26 cells in 200 μL PBS and treated i.p. from 3 days post inoculation three times at weekly intervals with CCMV (200 μg in 200 μL PBS), poly(I:C) (∼60 μg in 200 μL PBS), CCMV‐poly(I:C) (200 μg in 200 μL PBS), oxPt (5 mg kg^−1^) or CCMV‐poly(I:C) + oxPt (doses as above). We collected i.p. washes on day 24 and determined the level of cytokines and chemokines using an MSD assay using a customizable U‐PLEX analyzing the following cytokines/chemokines according to manufacturer's instructions: GM‐CSF, IFN‐α, IFN‐β, IFN‐γ, IL‐2, IL‐4, IL‐5, IL‐9, IL‐10, IL‐12p70, IL‐12/IL‐23p40, IL‐13, IL‐15, IL‐17A, KC/GRO, eotaxin, IP‐10, MCP‐1, MIP‐1α, MIP‐1β, TNF‐α, VEGF‐A, and RANTES. The plate was read using a MESO QuickPlex SQ 120 instrument and analyzed using MSD Workbench 4.0 software.

##### Flow Cytometry

The i.p. washes of CT26 tumor‐bearing mice were stained with Live/Dead aqua (1:1000; Thermo Fisher Scientific) and blocked with CD16/CD32 (1:1000; BioLegend) before staining for CD45 (pacific blue, 30‐F11), CD3ε (APC/Cyanine 7, 145‐2C11), CD8a (APC, 53‐6.7), and CD4 (FITC, GK1.5) (1:1000; BioLegend). The stained cells were analyzed using a BD FACSCelesta and a minimum of 10 000 cells was acquired. Data were analyzed using FlowJo software.

##### Enzyme‐Linked Immunosorbent Spot (ELISPOT) Assay

Spleens were harvested from CT26 tumor‐bearing mice on day 24 and processed using a spleen dissociation kit and gentleMACS dissociator (Miltenyi Biotec). Splenocytes (1 × 10^5^ cells) were seeded in 96‐well plates and co‐incubated with CTL‐Test medium (negative control), CT26 (1 × 10^5^ cells), 4T1 (1 × 10^5^ cells), and phorbol myristate acetate/ionomycin (PMA/Iono) (positive control), respectively. The level of IL‐4 and IFN‐γ was measured using a Mouse IFN‐γ/IL‐4 Double‐Color ELISPOT kit (ImmunoSpot) according to the manufacturer's instructions.

##### Histological Evaluation

When CT26 s.c. tumors had reached a volume of ∼30 mm^3^, mice were challenged i.t. three times with VLPs at weekly intervals. Tumors were harvested on day 24 and fixed with 4% formaldehyde (Thermo Fisher Scientific) before embedding in OCT compound. We then prepared 8‐μm sections and stained them with the terminal deoxynucleotidyltransferase dUTP nick end‐labeling (TUNEL) kit (Promega) and anti‐calreticulin (anti‐CRT) antibodies (Invitrogen, diluted 1:500) to observe apoptosis and immunogenic cell death (ICD), respectively. Nuclei were visualized with DAPI. Fluorescent images were acquired by a confocal microscopy and fluorescence intensities were quantified using ImageJ.

##### Statistical Analysis

Data were analyzed in GraphPad Prism v8 and are presented as means ± standard deviations. For multiple comparisons, statistical significance (*p* < 0.05) was determined by one‐way analysis of variance (ANOVA) with a post hoc Tukey's honest significant difference (HSD) test.

## Conflict of Interest

The authors declare the following competing financial interest(s): Dr. Steinmetz is a co‐founder of, has equity in, and has a financial interest in Mosaic ImmunoEnginering Inc. Dr. Steinmetz is a co‐founder of, and serves as manager of Pokometz Scientific LLC, under which she is a paid consultant to Mosaic ImmunoEngineering Inc., Flagship Labs 95 Inc., and Arana Biosciences Inc. The other authors declare no potential conflict of interest.

## Data Availability

The data that support the findings of this study are available from the corresponding author upon reasonable request.
